# Monthly Summary

**Published:** 1874-04

**Authors:** 


### MONTHLY SUMMARY.

---

*The Siamese Twins.*—These xiphopage monsters, Chang and Eng Bunker, were born in Siam, in 1811, and lived for many years on their plantation in North Carolina, where they died January 17. Chang suffered from lung disease, but the immediate cause of his death is supposed to have been cerebral blood-clot, and Eng is thought to have died, about two hours later, from fright.

At a meeting of the College of Physicians of Philadelphia, held on the 18th of February, Drs. Wm. H. Pancoast and Harrison Allen, the committee of the College entrusted with the post-mortem examination of the twins, presented a preliminary report of their dissection, which was in brief to the following effect:—

The twins were found to be united at the ensiform cartilage by a band about four inches long, and eight inches in circumference. Upon exploring this band through the abdominal cavity, it was found that in Eng the peritoneal cavity extended by a pouch into the band, and terminated there, a short distance beyond the median line; and in Chang two peritoneal prolongations or pouches were found, also extending into the band beyond the median line, and overlapping, one above and the other below the single peritoneal pouch of Eng.

In regard to the vascular connection it was found that on throwing coloured plaster injection into the portal circulation of Chang it flowed through the vessels of the upper part of the band into the portal vessels of Eng. The hypogastic arteries were found to run upward in each body into the band, and were lost in their way toward the common umbilicus in the anterior inferior surface of the middle of the band. Subsequently it was found that the two livers, which were supposed to be joined only by bloodvessels, were really one body, the parenchymatous tissue being continuous between them, so that when they were removed from the bodies and placed on the table they formed one mass. The so-called tract of portal continuity is, therefore, liver-tissue. Chang was supposed to be possessed of one more pouch than Eng. When the liver was removed, however, an upper hepatic pouch was found also proceeding from Eng, so that the band contained four pouches of peritoneum, besides liver-tissue.—*Med. News and Library.*

---

*Needle Swallowed by a Child—Removed Six Months afterwards from the Thigh.*—By B. Schermerhorn, M. D., of Buskirk's Bridge, N. Y.—Emma S., a child of fourteen months, was brought to me June 18, 1873, by its parents, who gave the following history. About six months ago the child swallowed an ordinary sewing needle which lodged in the throat, and was pushed downward with a swab in the hands of its Grandmother. From that time until about a month since it gave the child no trouble; since then it has been more or less worrisome and fretful, and during the last night before being brought to me it cried the whole time. Upon dressing the little one in the morning the mother's attention was drawn to a little elevation of the skin, like a pimple, upon the outer aspect of the left thigh, midway between the knee aud hip-joint. Thinking of the needle they came with the child to my office, and after laying open the cuticle I removed a needle an inch and a quarter in length, which lay with its eye to the surface.—*Med. News and Library.*

---

*A New Way of Gargling.*—At a recent meeting of the Clinical Society of London, Dr. Guinier showed, means of the laryngoscope, that it was practicable to allow any fluid to pass beyond the epiglottis, and to remain between that organ and the vocal cords during the time occupied by a long expiration. or as long as the patient can " hold his breath." Dr. Guinier used water in his demonstration : the vocal cords were plainly seen through the fluid, and the operator also ejected the latter through the nose. Although the sensibility of the soft palate and parts adjacent is known to be, comparatively speaking, inconsiderable, a general impression appeared to exist among the members that much practice would be required on the part of the patient to bring about so successful a result as that achieved in his case by Dr. Guinier. The following rules must be observed and practiced in the operation : (1) Raise the head slightly, (2) open the mouth to a moderate extent, (3) advance the chin and lower the jaw, (4) sound, or make an intention of sounding the double vowel œ. The author remarks that, as a consequence of these four movements carried on simultaneously, the pharynx is widened, the soft palate and uvula are raised, the base of ths tongue is depressed, and the liquid thus flows by virture of ite own weight into the laryngeal cavity.—*Med. and Surg. Reporter.*

---

*A new Operation for Cleft Palate.*—On the 22d of November, Sir William Fergusson, in operating on two patients for the closure of the opening in the hard palate after the cleft in the soft palate had been closed, adopted a modification of a procedure which is intended to increase the chances of success of the operation. Sir William remarked that in the so-called Langenbeck operation—that is, where muco-periosteal flaps are taken from the roof of the mouth and drawn towards the middle line —the proceeding is often unsuccessful from the fact that, after some time, the granulations which are thrown out on the upper surfaces of the displaced flaps contract and separate the union that may have taken place between the pared edges of the flaps. It is true, he observed, that some assert that bony matter is deposited on the upper surface, and that this diminishes the size of the aperture in the osseous palate. But, in demurring to this, Sir William said he thought it was hardly possible to strip off healthy periosteum from the subjacent bone. He proposed, therefore, as a remedy, that in addition to making the ordinary incision for the flaps, the hard palate should be split on each side of the opening with some sharp cutting instrument, and that the two pieces of bone should be pressed towards the middle line, and the pared edges of the soft tissues then be brought together. By this means the central opening would be closed, but two lateral apertures would be formed. But inasmuch as the lateral openings would be but half the size of the original central one, and as there would be more likelihood of the fractured edges of bone throwing out osseous material for its repair, it was hoped that the prospect of a successful issue would be greatly enhanced.

It remains to be seen what will be the result of this ingenious device, but on the first blush it appears that by its adoption a means is offered of surmounting one of the most obstinate difficulties of plastic surgery.—*Lancet.*

---

*New Source of India Rubber.*—Although it has long been known that the milky juice of the common silk-weed, *Asclepias Cornuti*, contains a proportion of caoutchouc, and though thirty years ago Schultz announced this constituent to exist in notable quantity, yet, until lately, we were not aware of any practical advantage having been taken of the fact. We understand, however, that, a short time ago, this subject attracted the attention of some gentlemen residing in the Western portion of this province, and that, on the strength of preliminary experiments, a company, having an authorized capital of $100,000, was formed at London, Ont., for the purpose of carrying out this new and promising branch of manufacture.

So far the undertaking has not passed the experimental stage, as the company are desirous of ascertaining the best methods of manufacture, and the probable extent of the business, before encroaching largely upon their capital. At the outset, a call of five per cent. was made, and the result of the experiments made was so encouraging as to warrant a further call of five per cent. for the purpose of continuing these experiments on a more extensive and practical scale.

In a late experiment, one thousand pounds of milk-weed were operated upon, and this quantity was said to yield about four per cent. of caoutchouc. The process consisted in subjecting the plant to partial decomposition, heating by steam, and then treating with coal-tar naptha. The benzine, holding the caoutchouc in solution, was then distilled, when the rubber was finally obtained in a solid form. Of the benzine, about eighty per cent. was recovered.

The rubber so obtained possesses all the ordinary characteristics of pure caoutchouc, and in its solubilities is identical with that substance. The company have, of course, protected their manufacture by patents; and we understand that in the United States, if not in Canada, patents for applying the process of vulcanization have also been obtained. The original patent does not apply exclusively to milk-weed, but is extended to other caoutchouc-bearing plants, as the bamboo-berry, flaxseed, etc.

A curious fact is stated in regard to the benzine recovered by distillation from the plant mentioned While, in its original condition, benzine is exceedingly inflammable and explosive, it is said that it loses the latter property after distillation, and, in regard to the former, is so modified as to be easily manageable; so much so, that the company intend offering it for sale as a new burning fluid. Perhaps some of our readers can account for this change of properties.—*Canadian Pharm. Journal.*

---

*Ergot in Epistaxis.*—Dr. Andrew H. Smith (*The Medical Record*, October 15, 1873) reports the case of a man, æt. 37, who suffered from persistent epistaxis, recurring sometimes two or three times a day. Direct and rhinoscopic examinations showed no abnormal condition of the nasal mucous membrane. Astringents were applied locally by means of both the brush and syinrge, and such general treatment was resorted to as the symptoms demanded. After this had been persevered in for two weeks without affecting the hemorrhages, the fluid extract of ergot was prescribed in twenty-drop doses three times a day.

This was continued for ten days, with the effect of entirely restraining the bleeding from the time the first dose was taken. The medicine was then omitted, but in a few days the bleeding began anew. It was immediately arrested by a resort to the drug, and did not afterwards return; the medicine being continued at gradually increasing intervals for nearly a month.

---

*Cure for Baldness.*—An English paper relates the following case:—

A gentleman, who had lost nearly all his hair after a very severe attack of fever, consulted a French physician of great reputed success as a hair-restorer. The prescription was a drachm of the homœopathic tincture of phosphorous to one ounce of castor oil; the bare spot to be rubbed with this mixture three times weekly for half an hour each time, after the skin of the head had been thoroughly cleansed with warm water without soap. This treatment was faithfully carried out for about six months; the hair soon began to grow, and in a year from the time of first following the doctor's advice, his head was as thoroughly covered as ever, the new crop of hair being about two shades darker than the old.—*Druggists' Circular.*

